# Parent Engagement With a Telehealth-Based Parent-Mediated Intervention Program for Children With Autism Spectrum Disorders: Predictors of Program Use and Parent Outcomes

**DOI:** 10.2196/jmir.4913

**Published:** 2015-10-06

**Authors:** Brooke Ingersoll, Natalie I Berger

**Affiliations:** ^1^Department of PsychologyMichigan State UniversityEast Lansing, MIUnited States

**Keywords:** autism, telehealth, parent training

## Abstract

**Background:**

There has been growing interest in using telehealth to increase access to parent-mediated interventions for children with ASD. However, little is known about how parents engage with such programs.

**Objective:**

This paper presents program engagement data from a pilot study comparing self-directed and therapist-assisted versions of a novel telehealth-based parent-mediated intervention for young children with autism spectrum disorders (ASD).

**Methods:**

Parents of young children with ASD were randomly assigned to receive a self-directed or therapist-assisted version of ImPACT Online. Parent engagement and satisfaction with the different components of the program website were examined using the program’s automated data collection and a post-treatment evaluation survey. We examined the relationship between program engagement and changes in parent knowledge and implementation and participant characteristics associated with program engagement.

**Results:**

Of the 27 parent participants, the majority were female (26/27, 96%), married (22/27, 81%), with a college degree or higher (15/27, 56%), and less than half were not employed outside of the home (10/27, 37%). The mean chronological age of the child participants was 43.26 months, and the majority were male (19/27, 70%) and white (21/27, 78%). Most of the families (19/27, 70%) resided in a rural or medically underserved area. Parents logged into the website an average of 46.85 times, spent an average of 964.70 minutes on the site, and completed an average of 90.17% of the lesson learning activities. Participants in the therapist-assisted group were more likely to engage with the website than those in the self-directed group: *F*_2,24_=17.65, *P*<.001. In total, 85% of participants completed the program, with a significantly greater completion rate in the therapist-assisted group (N=27): χ^2^_1_=5.06, *P*=.03. Lesson learning activities were visited significantly more often than the supplemental activities (all *P*s<.05). Multiple regression controlling for pretreatment performance indicated that program completion (beta=.51, *P*=.02) predicted post-treatment intervention knowledge, and program completion (beta=.43, *P*=.03) and group assignment (beta=-.37, *P*=.045) predicted post-treatment intervention fidelity. Partial correlations indicated that parent depressive symptoms at pretreatment were negatively associated with program completion (*r*=-.40, *P*=.04), but other key parent and child demographic factors were not. Post-treatment measures of website usability (*r*=.65, *P*<.001), treatment acceptability (*r*=.58, *P*=.002), and overall satisfaction (*r*=.58, *P*=.002) were all related to program completion.

**Conclusions:**

Parent engagement and satisfaction with ImPACT Online was high for both self-directed and therapist-assisted versions of the program, although therapist assistance increased engagement. Program completion was associated with parent outcomes, providing support for the role of the website in parent learning. This program has the potential to increase access to parent-mediated intervention for families of children with ASD.

## Introduction

The past 20 years have seen a dramatic increase in the proliferation of telehealth—the delivery of health and mental health information via the Internet and related technologies. There are numerous potential advantages of the use of telehealth to augment or even replace traditional service delivery models. Perhaps most appealing is its potential to increase access to evidence-based interventions for individuals living in rural and underserved areas at a significantly reduced cost [1]. Such programs have been shown to greatly improve care for patients with chronic diseases, such as diabetes, heart disease, and asthma [2-4], and increase access to evidence-based health promotion [5-7], psychological [8-10], and parenting interventions [11]. Patients are often very satisfied with the care they receive through telehealth services [12,13], and both efficacy and effectiveness trials have found moderate to large effects of telehealth interventions on participant knowledge and behavior [14-16].

Telehealth’s ability to increase access to services in a cost effective manner [1] indicates that it may be an attractive option for the treatment of autism spectrum disorders (ASDs). Given that children with ASD experience significant difficulties accessing traditional services [17] and that the prevalence rate of ASD has increased significantly in the past 15 years [18], alternative service delivery models are necessary in order to provide this unique population with sufficient treatment options. Indeed, survey-based studies have indicated that approximately one third of children with ASD have problems obtaining specialty care [19], and approximately 40% experience difficulties accessing desired services more generally [20]. Difficulty accessing services is compounded for children with ASD from underserved areas [21] and those belonging to racial and ethnic minority families [17,20].

There has been growing interest in the use of telehealth for delivering parent-mediated interventions for children with ASDs [22-26]. Children with ASD experience pervasive deficits in social communication and the presence of restricted and repetitive behaviors [27], which can significantly impact the quality of life of the child and family [28-30]. Parent-mediated intervention, in which the parent is trained to use intervention techniques directly with their child, has been widely recognized as a critical component of effective autism intervention [31,32]. Research indicates that parents can learn to use evidence-based intervention techniques with fidelity [33-36], and parent use of the techniques results in improvements in child skills and behavior [34,37]. Further, parent-mediated intervention is associated with improvements in family quality of life [38,39]. Despite its benefits, parent-mediated intervention is highly under-utilized in community settings [40]. Barriers include a shortage of trained professionals, lengthy waitlists, limited financial resources and transportation, lack of child care, geographic isolation, and time limitations [41-43], many of which are compounded in families who live in rural and underserved areas [44].

Although the evidence base at this point is very limited, several quasi-experimental and single-case design studies have demonstrated the potential of telehealth to deliver parent-mediated intervention for children with ASD. Parents generally find such programs to be acceptable [26], and program use is associated with gains in parent knowledge [45-48] and use of evidence-based intervention procedures [26,36,49]. Importantly, there is also emerging evidence for improvement in child social communication skills as a result of parent use of such programs [23,36,49].

These studies are encouraging and suggest that telehealth, should it prove efficacious in larger trials, may be an effective method for increasing access to parent-mediated interventions for families of children with ASD. However, there is very little known about factors that influence parent engagement with such programs. For example, it is not clear what types of parents choose to enroll in telehealth-based parent-mediated intervention programs and whether they are different from parents who enroll in traditional parent-mediated intervention programs. It is also unclear whether specific supports provided during the program influence program engagement. Several studies have suggested that therapist assistance can positively influence participant engagement with cognitive-behavioral therapy (CBT)-based telehealth interventions for mood and anxiety [10], and remote coaching by a therapist after participation in a self-directed, telehealth-based parent-mediated intervention was shown to improve parents’ ability to implement an imitation intervention with their child with ASD [26]. However, the role of therapist assistance in promoting program engagement with telehealth-based parent-mediated intervention programs for ASD is unknown. Further, although research has indicated that parent participation in such programs is associated with gains in parent knowledge and skill use [23,50], additional research is needed to examine to what extent parent engagement with the website used to deliver programs is related to these important parent outcomes.

Finally, little is known about parent and/or child characteristics that are associated with program engagement in telehealth-based parent-mediated intervention. Across a range of health information technologies, participants’ computer/Internet self-efficacy, as well as the perceived usefulness and ease of use of the technology have been associated with technological acceptance [51], suggesting that these factors may influence parents’ participation in telehealth-based parent-mediated intervention programs. However, the majority of these studies examined behavioral intention or self-reported use, rather than actual use or engagement with the technology, and studies that have used objective measures of program use have sometimes failed to find a relationship with these variables [52,53]. Treatment acceptability, or the degree to which consumers view an intervention as appropriate, fair, and reasonable for the presenting problem [54], has been associated with client engagement in both behavioral and medical treatments [55,56]; however, little research has examined the relationship between treatment acceptability and engagement in telehealth-based interventions [57]. Sociodemographic factors, such as age, gender, race/ethnicity, marital status, education, and employment, have often failed to emerge as consistent predictors of engagement in telehealth programs more broadly [51]. At the same time, these factors have been found to influence engagement in traditional parent-mediated intervention programs, with lower socioeconomic status and maternal mental health problems being particularly salient predictors of dropout [58]. It is not yet known whether these same characteristics apply to telehealth-based parent-mediated intervention for children with ASD. This is a particularly important area to examine, given that lower socioeconomic status is associated with significantly poorer access to services for children with ASD [17,21].

In this paper, we present data about program engagement from a pilot study of ImPACT Online, a telehealth-based parent-mediated intervention for young children with ASD. Specifically, we examined (1) the demographics of parent and child participants who enrolled in the program, (2) the overall level of program engagement among parents of children with ASD, the components of the website that parents used most and least often, and parents’ perceptions of the usefulness of the different program components, (3) whether therapist assistance influenced engagement with or perceived usefulness of the program, (4) whether parent engagement with the website was associated with changes in parent intervention knowledge and intervention fidelity, and (5) whether pretreatment participant characteristics (parent computer/Internet fluency, parent depressive symptoms, family demographics, child severity) and/or parents’ experience with the program (website usability, treatment acceptability, overall satisfaction) were associated with greater engagement with the website.

## Methods

### Participants

Participants included 28 parents of a child with ASD between the ages of 27 and 73 months. Participating families were recruited from agencies serving children with ASD (eg, early intervention programs, diagnostic centers, parent support groups) and online. Recruitment focused on underserved communities as defined by residence in a rural (based on the United States Department of Agriculture Rural Development designation) and/or medically underserved area/provider shortage area (based on the Health Resources and Services Administration medically underserved area and population designation). All children met criteria for Autistic Disorder or Pervasive Developmental Disorder, Not Otherwise Specified based on the *Diagnostic and Statistical Manual – 4th Edition, text revision* (DSM-IV-TR) criteria [59] and the Autism Diagnostic Observation Schedule, generic or 2nd edition (ADOS-G/ADOS-2) [60]. Parents had to be proficient in English, although other languages could be spoken in the home. For two-parent households, both parents were able to use the online tutorial if they wanted; however, only 1 parent participated in data collection and received online coaching (for the therapist-assisted group).

Families without a personal computer, webcam, or high-speed Internet in the home were provided the required technology. One family suspended treatment for 7 months in the middle of the program due to a significant health problem. This family’s data were excluded from analysis as their pre-post data were not comparable to the other families’, yielding a total of 27 participants. All parents gave informed consent for their own and their child’s participation under the oversight of Michigan State University’s Human Research Protections Program. Table 1 presents participant characteristics by group.

**Table 1:** Participant demographic information.

		Overall	Group		Test statistic	*P* value
			Self-directed	Therapist-assisted		
**Parent characteristics**						
	Gender (% female)	96	92	100	1.12^a^	.29^a^
	Education level (% college degree)	56	46	64	0.90^a^	.34^a^
	Marital status (% married)	81	92	71	1.95^a^	.16^a^
	Employment status (% not employed)	37	46	29	0.65^a^	.42^a^
	Residence in underserved area, %	70	77	64	0.52^a^	.47^a^
	Computer/Internet self-efficacy (CEWFS)	35.57 (4.25)	35.09 (4.76)	36.00 (3.89)	0.50^b^	.62^b^
	Depressive symptoms (CES-D)	10.89 (8.26)	10.23 (6.98)	11.50 (9.52)	0.39^b^	.70^b^
**Child characteristics**						
	Gender (% male)	70	61	79	0.94^a^	.33^a^
	Race/Ethnicity (% white)	78	92	64	3.06^a^	.08^a^
	Chronological age in months, mean (SD)	43.26 (12.58)	46.08 (13.18)	41.57 (12.24)	-0.71^b^	.48^b^
	Nonverbal mental age in months, mean (SD)	24.83 (11.57)	25.42 (13.92)	24.29 (9.38)	-0.25^b^	.80^b^
	Verbal mental age in months, mean (SD)	20.44 (10.11)	19.15 (9.63)	21.64 (10.74)	0.63^b^	.53^b^
	ADOS comparison score, mean (SD)	7.00 (1.61)	7.00 (1.87)	7.00 (1.35)	0.00	˃.99^b^
	Nonstudy intervention hrs/wk, mean (SD)	12.98 (10.15)	13.62 (10.96)	12.38 (9.70)	-0.31^b^	.76^b^

^a^Chi square.

^b^ *t* test.

### Design Overview

Participating families provided demographic information and completed a standardized battery of assessments of parent and child functioning in the lab and family home at intake. Children were matched on expressive language on the Mullen Scales of Early Learning [61] and then randomly assigned to the self-directed or therapist-assisted group using a coin flip. At post-treatment, parents completed measures of parent learning, a survey-based evaluation of ImPACT Online, along with other measures of parent and child functioning (not presented here).

#### Interventions

##### Self-Directed Group

Parents assigned to the self-directed group received access to the secure, password-protected ImPACT Online website for up to 6 months. The program was adapted from Project ImPACT, which is a naturalistic, developmental-behavioral, parent-mediated intervention for young children with ASD [62]. The website contained 12 self-directed lessons, each of which took approximately 80 minutes to complete. Parents were encouraged to complete one lesson per week and to practice the intervention techniques with their child between each lesson. Each lesson included six learning activities that were designed to be completed in order. The topic was introduced via a Narrated Slideshow with embedded video clips of an expert therapist or parent walking step-by-step through each technique and providing tips for successful implementation. The same information was elaborated on in a Written Manual that could be printed for later reference. After each slideshow, parents completed a Self-Check Quiz and short Interactive Exercises, in which they viewed short video clips of adult-child interactions and were asked to identify accurate use of the intervention techniques. Parents were provided with immediate feedback on their performance to facilitate learning. At the end of the lesson, parents completed a Homework Plan in which they identified child goals, activities in which they would practice, and how they would implement the specific techniques within those activities. After completing the homework plan, parents responded to Reflection Questions that asked them to report how they used the techniques, their child’s response, what aspects went well, and what aspects were challenging.

Parents could also access supplemental material outside of the weekly lessons. Supplemental material included a Video Library that contained longer video examples of adults using all of the intervention techniques together, a Resources page with links to autism informational websites, and a moderated Forum that allowed parents to share information with other parents. Parents received weekly “Tip of the Week” emails that provided tips for implementing the intervention techniques along with a link to the program to encourage program use. Parents in the self-directed group were able to contact project staff via phone or email for assistance with technology-related problems (eg, difficulty with logins, problems playing video) but were given no assistance or support in learning the intervention from project staff outside of the self-directed website. See Multimedia Appendix 1 for screenshots.

##### Therapist-Assisted Group

Parents assigned to the therapist-assisted group were given access to the ImPACT Online website and were encouraged to work through the program at the same pace as the self-directed group. Parents also received two 30-minute remote coaching sessions per week (24 total sessions) via Skype video conferencing software by a trained therapist to assist them in learning the intervention. Skype was selected as the primary video conferencing software during the development phase of the program based on feedback from focus groups with parents and providers indicating that parents would be most comfortable using Skype (over other available systems) based on its simplicity and familiarity. Parents in this study were made aware that Skype was not Health Insurance Portability and Accountability Act (HIPAA)-compliant during the consent process.

The first coaching session of the week involved the coach and parent and was used to help clarify the content of the relevant lesson and help the parent apply the information to their child. The second coaching session of the week involved the coach, parent, and child and was used to provide the parent with “live” feedback on their use of the intervention techniques as they practiced with their child.

#### Measures

##### Participant Characteristics

Parents provided demographic information about themselves and their child at intake. Parent demographic variables included gender, marital status, education level, employment status, and residence in a rural and/or medical professional shortage area. Child demographic variables included age, gender, and race/ethnicity. Parents also provided information on the type and hours per week of all nonstudy treatments (eg, speech-language therapy, therapeutic preschool) their child received. Children were administered the Mullen Scales of Early Learning [61] by the study team to determine their developmental age and the ADOS-G or ADOS-2 [60] to determine autism severity.

##### Computer and Internet Fluency

Parents completed a modified version of the Computer-Email-Web Fluency Scale (CEWFS) [63] at intake to assess their comfort with computers and Internet technology. Items such as (1) How frequently do you conduct a search using an Internet search engine? and (2) How frequently do you send or receive email? were rated on a 5-point scale from never (1) to daily (5). In addition, parents were asked to indicate on a 7-point scale how many hours per week they used the Internet. Scores could range from 8-42, with higher scores indicative of greater fluency. Cronbach alpha for the modified scale was .70, indicating adequate internal consistency. Data were missing on the CEWFS for 4 participants.

##### Depressive Symptoms

Parents completed the Center for Epidemiological Studies-Depression Scale (CES-D) [64] at intake as a measure of depressive symptoms. Respondents used a 4-point scale to rate how often they experienced 20 different symptoms of depression over the past week, with higher scores indicating a greater degree of depressive symptomatology.

##### Intervention Knowledge

Parents completed the ImPACT Knowledge Quiz. This 20-item, multiple-choice quiz measures comprehension of the key elements of Project ImPACT, at intake and post-treatment to measure changes in their intervention knowledge. Data were missing at post-treatment for one participant.

##### Intervention Fidelity

Parents were videotaped during a parent-child interaction in their home at intake and post-treatment to measure changes in their intervention fidelity. Parents were asked to play with their child for 10 minutes and have a small snack or meal with their child. Parent behavior was scored for correct use of the intervention strategies using the Project ImPACT intervention fidelity checklist [62]. Each of the six fidelity dimensions was scored on a scale of 1 (Parent does not implement throughout session) to 5 (Parent implements throughout session) and then averaged to form an overall fidelity rating for each routine. The fidelity dimensions included (1) Focus on your child, (2) Adjust your communication, (3) Create opportunities for engagement, (4) Teach your child new communication skills, (5) Teach your child new play skills, and (6) Pace the interaction. Ratings for the play and snack routines were averaged to form an overall fidelity rating for each time point. Data were missing at post-treatment for one participant.

##### Program Engagement

Program engagement was calculated from the ImPACT Online website’s electronic tracking of user behavior. Several different metrics of program engagement were calculated, including (1) average number of logins to the site, (2) average duration of time spent on the site across the intervention period, and (3) program completion, which was defined as completion of at least 75% of total learning activities across the 12 lessons (out of a possible 71). In addition, we examined the average duration of each login, the average number of days to program completion, and the times of day when logins occurred.

To better understand how parents used the program, we calculated the average number of visits made to each of the lesson learning activities (slideshow, manual, self-check, exercises, homework, reflection) and supplemental materials (video library, resources, forum), as well as the percent of each type of lesson learning activity completed.

##### Program Evaluation

At post-treatment, parents were asked to complete an evaluation survey measuring treatment acceptability, website usability, and overall program satisfaction. All items were rated using a 7-point Likert scale, with higher scores indicative of greater satisfaction. Parents rated the acceptability of the treatment using a 30-item questionnaire [65] adapted from the Treatment Evaluation Inventory [66] and the Behavior Intervention Rating Scale [67]. Cronbach alpha for the treatment acceptability measure was .93. Parents rated the usability of the website using an 11-item questionnaire developed for this project. For this measure, parents rated the perceived helpfulness of each program component using the following statement: “The [individual program component]s were helpful for learning the ImPACT Online intervention”, as well as 3 additional items: (1) “It was easy to find information on the program website,” (2) “I used the website to complete the skills-training sessions,” and (3) “I understood the audio and text information that was presented.” Cronbach alpha for the website usability scale was .81. The average of two additional items, (1) “I used the intervention with my child regularly” and (2) “I would recommend this program to other parents of young children with social-communication difficulties, was used as a measure of overall program satisfaction.”

Parents in the coaching group were also asked to rate the quality of the therapist relationship using the following items: (1) “My coach was interested in me,” (2) “My coach understood me,” and (3) “My coach understood my child, and the perceived helpfulness of (i) the weekly discussion sessions with the trainer, (ii) the weekly coaching sessions with my child.”

## Results

The data were examined for normality using visual analysis of the data distributions and the inspection of the skewness coefficient. All variables were found to be normally distributed with the exception of overall satisfaction, which was subject to ceiling effects. Thus, we used nonparametric tests (Mann-Whitney U, Spearman rank correlation) for analyses involving the overall satisfaction variable and parametric tests for all other analyses.

### Participant Characteristics

As shown in Table 1, the majority of parent participants were female (26/27, 96%), married (22/27, 81%), with a college degree or higher (18/27, 66%). Less than half of parents were not employed outside of the home (10/27, 37%). The majority of child participants were male (19/27, 70%) and white (19/27, 78%). The children’s mean chronological age was 43.26 months. All children exhibited a significant developmental delay, with an average nonverbal mental age of 24.83 and verbal mental age of 20.44, and their average autism severity fell in high moderate range. These characteristics are relatively typical of families who participate in parent-mediated intervention studies for young children with ASD [68]. Consistent with our effort to recruit families in underserved areas, 70% (21/27) of our sample resided in a rural or medical underserved area. Two parents requested technology (2 needed a computer with webcam and 1 needed high-speed Internet) in order to be able to complete the program in their home. Scores on the CEWFS ranged from 26 to 40, with average scores toward the upper end of the scale (35.8), suggesting that most parents felt fluent with computer and Internet technology. The majority of parents reported using computers 10 or more hours per week, although 22% reported using computers 4 hours a week or less. Parents reported that their children received a variety of different intervention services (eg, special education, applied behavior analysis, speech therapy, occupational therapy, play groups), and the number of hours of nonstudy intervention they received per week ranged from 0.5 to 38.5 hours. We used independent sample *t* tests and chi-square tests as appropriate to examine group difference on pretreatment characteristics. There were no significant between-group differences on participant characteristics at intake.

### Program Engagement

As can be seen in Table 2, there was a high rate of program engagement as measured by the various metrics. Parents logged into the website an average of 46.85 times and spent an average of 964.70 minutes on the site over the intervention period. Eighty-five percent of parents (23/27) completed the program—a completion rate that is similar to the traditional, in-person version of the program [69]. Parents averaged 21.23 minutes per login and took an average of 133.87 days to complete the program. Parent logins were equally likely to occur throughout the day (8-11 a.m.: 19.67%; 11 a.m.-2 p.m.: 15.90%; 2-5 p.m.: 19.28%) and evening (5-8 p.m.: 17.52%; 8-11 p.m.: 19.70%)]. Parents logins were significantly less likely in the early morning (5-8 a.m.: 4.45%) and overnight (11 p.m.-5 a.m.: 3.48%). It should be noted that almost half of all logins (45.19%) occurred between 5 p.m. and 8 a.m., which are times of day when traditional therapy is typically not available.

We examined group differences on the two continuous metrics of program engagement (number of logins, duration on site) using a multivariate analysis of variance (MANOVA), and program completion using chi-square. There was a significant effect of group on program engagement: *F*_2,24_=17.65, *P*<.001. Follow-up tests revealed that the therapist-assisted group demonstrated significantly greater program engagement on both metrics than the self-directed group (see Table 2). The therapist-assisted group was also significantly more likely to complete the program than the self-directed group (N=27): χ^2^_1_=5.06, *P*=.03.

**Table 2:** Program engagement.

		Overall, mean (SD)	Self-directed, mean (SD)	Therapist-assisted, mean (SD)	Test statistic	*P* value
Number of logins		46.85 (22.30)	29.54 (13.76)	62.93 (15.55)	34.68	<.001
Duration of time on site		964.70 (518.49)	707.04 (402.41)	1203.94 (510.06)	7.81	.01
Participants completing program, %		85	69	100	5.06	.03
Days to completion		133.87 (38.30)	148.56 (41.17)	124.42 (34.55)	-1.52	.14
Duration per login		21.23 (6.70)	23.38 (4.91)	19.23 (7.66)	-1.66	.11
**Visits to lesson learning activities**						
	1. Slideshows	18.22 (6.50)^a^	16.54 (4.99)	19.79 (7.48)	1.32	.20
	2. Manual	23.59 (11.40)^a^	21.15 (9.02)	25.86 (13.17)	1.07	.29
	3. Self-check	14.56 (4.61)^b^	12.69 (4.25)	16.29 (4.38)	2.16	.04
	4. Exercises	14.15 (5.11)^b^	11.62 (4.63)	16.50 (4.50)	2.79	.01
	5. Homework	31.93 (19.54)^c^	22.46 (10.85)	40.71 (21.95)	2.70	.01
	6. Reflection	20.78 (9.17)^a^	16.69 (9.69)	24.57 (7.02)	2.43	.02
**Visits to supplemental materials**						
	7. Video library	4.04 (3.45)^d^	2.23 (1.79)	5.71 (3.81)	3.00	.01
	8. Resources	1.85 (2.32)	1.69 (2.90)	2.00 (1.71)	0.34	.74
	9. Forum	2.33 (1.82)^d^	2.54 (2.03)	2.14 (1.66)	-0.56	.58

^a^Significantly greater than 3, 4, 7, 8, 9.

^b^Significantly greater than 7, 8, 9.

^c^Significantly greater than 1, 2, 3, 4, 6, 7, 8, 9.

^d^Significantly greater than 8.

We then compared the average number of visits parents made to each program component (ie, learning activities and supplemental materials) using a mixed-model, repeated-measures ANOVA with group as a between-subjects variable and program component as a within-subjects variable. There was a main effect of group (*F*_1,25_=241.86, *P*<.001) favoring the therapist-assisted group, and a main effect of program component (*F*_8,200_=57.66, *P*<.001). Follow-up paired *t* tests indicated that parents were most likely to visit the homework, followed by the reflection, manual, and slideshow, and then the self-check and exercises. Parents visited each of the lesson learning activities more often than the supplemental materials (all *P*s≤.05). There was also a significant group x program component interaction. Follow-up independent samples *t* tests indicated that the therapist-assisted group visited the self-check, exercises, homework, reflection, and video library significantly more than the self-directed group (all *P*s<.05). However, the groups did not differ in number of visits to the slideshow, manual, resources, and forum.

The number of visits to each learning activity contained in the lessons may be slightly misleading as a metric of engagement, as the different activities may pull for a different number of visits. For example, parents may only visit the self-check questions once in order to assess their comprehension, whereas they might return several times to the homework page in order to complete or review their homework plan. Therefore, we also examined the percent of learning activities that parents completed, regardless of the number of visits that this took. Again, there was a main effect of group (*F*_1,25_=4.37, *P*=.047) favoring the therapist-assisted group, and a main effect of learning activity (*F*_5,125_=3.33, *P*=.007). But there was not a group x learning activity interaction (**P*>.*05). Follow-up paired *t* tests indicated that parents completed the reflection (mean 83.8%, SD 5.70%) significantly less often than each of the other lesson learning activities (range 87.65-92.90%; all *P*s<.05), with the exception of the slideshow for which the difference was marginal (*P*=.095). There was no difference in completion rates between any of the other learning activities.

### Program Evaluation

Parents rated the intervention content as highly acceptable (mean 6.07, SD 0.79) and the website as highly usable (mean 6.36, SD 0.57). In addition, parents indicated that they were highly satisfied with the program overall (mean 6.56, SD 0.71). The therapist-assisted group rated the quality of the therapist relationship uniformly highly (mean 6.99, SD 0.06). There were no group differences on ratings of treatment acceptability (*t*_25_=1.79, *P*=.09) or website usability (*t*_25_=1.28, *P*=.21). There was a marginally significant group difference on parents’ rating of overall program satisfaction, with parents in the therapist-assisted group rating the program significantly higher (mean 6.86, SD 0.23) than those in the self-directed group (mean 6.23, SD 0.90; *Z*=1.92, *P*=.054).

### Effect of Program Engagement on Parent Outcomes

Next, we examined the degree to which program engagement was predictive of parent intervention knowledge and intervention fidelity at post-treatment. We chose to focus on program completion (ie, completion of at least 75% of all lesson learning activities) because it is a conservative indicator of exposure to program content across intervention delivery modalities [70]. For these analyses, we employed a series of linear regressions, with parents’ intervention knowledge or intervention fidelity at post-treatment as the dependent variable, and parents’ pretreatment score for the relevant measure, group assignment, and program completion as independent variables.

The full model predicting parents’ post-treatment intervention knowledge was statistically significant: *F*_3,25_=4.81, *P*=.01. After controlling for pretreatment intervention knowledge, program completion was a significant predictor of post-treatment intervention knowledge: beta=.45, *t*=2.45, *P*=.02 (Table 3). This finding suggests that regardless of group assignment, parents who completed the program experienced more improvement in their intervention knowledge from pre- to post-treatment. The full model predicting parents’ post-treatment intervention fidelity was also significant: *F*_3,25_=5.87, *P*=.004. After controlling for pretreatment intervention fidelity, both group assignment (beta=-.37, *t*=-2.12, *P*=.045) and program completion (beta=.43, *t*=2.30, *P*=.03) were significant predictors of intervention fidelity at post-treatment. This result indicates that both therapist assistance and program completion make independent contributions to gains in parents’ accurate use of the intervention techniques with their child.

**Table 3:** Predictors of parent intervention knowledge and parent intervention fidelity at post.

	Post-treatment performance			
	Parent knowledge		Parent fidelity	
Predictors	Beta	*t*	Beta	*t*
Pretreatment performance	0.40	2.39^a^	0.38	2.21^a^
Group assignment	0.04	0.21	-0.37	-2.12^a^
Program completion	0.51	2.81^a^	0.43	2.30^a^

^a^ *P*<.05.

### Predictors of Program Engagement

Finally, we examined the extent to which parent and child demographic factors at pretreatment and parent program evaluation variables at post-treatment were associated with program engagement. Again, we focused on program completion as our measure of program engagement. To do this, we calculated partial correlations between participant demographic variables at intake, program evaluation variables at post-treatment, and program completion, controlling for group assignment. Parent demographic variables included education level (no college degree vs college degree or higher), marital status (married vs not married), employment status (employed full or part time vs not employed), residence in underserved area, computer/Internet self-efficacy, and depressive symptoms. We did not examine parent gender as there was only 1 male parent participant. Child demographic variables included age, gender, race/ethnicity (majority vs minority), developmental quotient, autism severity (ADOS comparison score), and hours of nonstudy intervention. Program evaluation measures included treatment acceptability, website usability, and overall program satisfaction.

Of all of the pretreatment parent and child demographic factors, only parent depression was significantly associated with program completion: *r*_24_=-.42, *P*=.04 (Table 4). Parents who rated themselves higher in depressive symptoms at pretreatment were significantly less likely to complete the program. All program evaluation variables (treatment acceptability: *r*_24_=.58, *P*=.002; website usability: *r*_24_=.65, *P*<.001; and overall program satisfaction: *r*_24_=.58, *P*=.002) were significantly associated with program completion.

**Table 4:** Partial correlates of program completion controlling for group assignment.

		Program completion
**Parent**		
	Education level	0.20
	Marital status	-0.10
	Employment status	0.04
	Residence in underserved area	-0.24
	Computer/internet self-efficacy	0.16
	Depressive symptoms	-0.40^a^
**Child**		
	Age	0.13
	Gender	-0.12
	Race/Ethnicity	0.09
	Developmental quotient	0.10
	Autism severity	-0.15
Hours of nonstudy intervention		0.24
Website usability		0.65^c^
Treatment acceptability		0.58^b^
Overall program satisfaction		0.58^b^

^a^ *P*<.05.

^b^ *P*<.01.

^c^ *P*<.001.

## Discussion

### Principal Findings

In this study, we examined parent engagement with a novel, telehealth-based parent-mediated intervention for children with ASD. Overall findings indicated that parent engagement and satisfaction with ImPACT Online was high for both self-directed and therapist-assisted versions of the program, although therapist assistance increased engagement. Parent outcomes were associated with program completion, providing support for the role of the website in parent learning.

Parents who enrolled in this study were quite similar to participants in studies of traditional parent-mediated interventions for children with ASD [68]. Like most of these studies, the majority of our parent participants were married, college-educated mothers who were not employed outside of the home, and our child participants were white males, with a moderate ASD and significant developmental delay. However, unlike many previous studies, the majority of our sample lived in a rural or medically underserved area or were a medically underserved population. This suggests that telehealth-based parent-mediated intervention appeals to similar families who seek out traditional parent-mediated intervention (at least those who are involved in research) and may be able increase access to families in underserved areas. At the same time, there were few very low socioeconomic status families in our sample, and thus participation was not representative of the range of families who could potentially benefit from this type of intervention. Thus, future research should examine the potential reach of telehealth-based parent-mediated intervention in the population of families of young children with ASD as well as the representativeness of parents who enroll in these interventions.

We found high rates of program engagement among parents, with overall completion rates similar to those found in studies of traditional parent-mediated intervention programs for children with ASD [71,72]. Parents also rated the program very highly in terms of the acceptability of the treatment, the usability of the website, overall satisfaction with the program, and the quality of the therapist relationship (for the therapist-assisted group). Many of the parents engaged with the website during times of day when traditional parent training would not occur (eg, early mornings, evenings, weekends). Our data suggest that placing instructional activities online does not compromise parent engagement or parent satisfaction with parent-mediated intervention and may allow families to learn during times that are more convenient, such as before or after work and when children are in bed.

Parents were more likely to engage with certain program components than others. Specifically, the learning activities contained in the lessons were visited significantly more often than supplemental materials. Among these learning activities, parents were significantly less likely to complete the reflection than the other learning activities. Interestingly, the forum was rarely visited and only three parents posted to the forum even once. Models of technological acceptance that claim the perceived usefulness and ease of use a technology (or in our case, component) have a strong influence on its use [73]. This finding suggests that adjustments could be made to those components that were used less often to increase their perceived usefulness and/or ease of use.

Therapist assistance positively influenced parent engagement with the program. This finding is consistent with research on telehealth-based behavioral interventions; therapist support increases engagement with the program as well as overall outcomes [10,74]. In our study, therapist assistance was associated with a higher rate of program completion, as well as more frequent logins and a greater duration of time spent on the site. Therapist assistance appeared to be particularly beneficial for encouraging parents to visit specific components of the program, namely the self-check questions, exercises, homework, reflection, and video library. It may be that these components are more challenging or less motivating for parents to engage with on their own. Alternately, it may be that parents accessed these components during their coaching sessions after having completed them on their own. For example, parents were encouraged to go over their homework plan and reflection with their coach. The fact that therapist assistance did not seem to differentially affect the specific learning activities that were completed supports this possibility and suggests that a greater understanding of how therapist assistance influences parents’ use of the different program component would be important. Therapist assistance did not affect parents’ overall perception of acceptability of the treatment or the usability of the website. It did, however, marginally impact the parents’ overall satisfaction with the program. This greater level of satisfaction may have impacted parents’ willingness to engage with the website.

Parent completion of the learning activities on the website was a significant predictor of gains in parent knowledge and intervention fidelity. This finding suggests that program engagement was associated with gains in both conceptual and procedural knowledge of the intervention regardless of group assignment. Our regression model indicated that group assignment was also a significant predictor of gains in parent fidelity, which suggests that therapist assistance provided an additional benefit above and beyond completion of the tutorial for procedural knowledge. This finding is consistent with a large body of literature on parent training, which suggests that coaching is an important component for improving parent use of intervention techniques [43,49,75,76].

We examined a number of pretreatment parent and child demographic variables as potential predictors of parent program engagement. Only one of these variables, parent depressive symptoms, was significantly associated with program completion. Parents who reported more depressive symptoms at intake were less likely to complete the program than parents who reported fewer depressive symptoms. This finding is consistent with research on traditional parent training that has found that pretreatment parent psychopathology, particularly depression, increases the likelihood of attrition [58,77,78]. Thus, future studies should aim to examine the role of depression as a moderator of treatment effects for telehealth-based parent-mediated intervention. Our failure to find an association between program engagement and sociodemographic factors may have been due to our small sample size, and rather homogenous participant characteristics. However, a number of highly powered studies have failed to find an association between sociodemographic factors and use of health information technology [52,53,79]. Surprisingly, we did not find an association between program engagement and computer/Internet self-efficacy. It may be that computer/Internet self-efficacy may be more likely to affect a parent’s choice to enroll in a telehealth-based parent-mediated intervention program rather than to engage with the program once enrolled. Indeed, overall levels of computer/Internet self-efficacy in this sample were relatively high, and almost all participants had access to a computer with Internet in their home prior to study enrollment. Thus, research is needed to determine uptake and engagement with telehealth-based parent-mediated intervention among parents with lower computer/Internet self-efficacy and those with limited computer and Internet access.

In contrast, all of the post-treatment evaluation items were related to program engagement. The relationship between these factors and engagement is complex. Perceived usefulness and ease of use have consistently been found to predict behavioral intention to use a new technology and are considered important antecedent determinants of technological acceptance [80,81]. At the same time, engagement with a new technology can influence its perceived usefulness and ease of use [82]. Similarly, treatment acceptability has been found to predict engagement in intervention [83]; however, it can also be affected by intervention participation [84]. As these measures were taken post-treatment in our study, the direction of influence is not clear. However, these findings highlight the importance of participants’ experience with both the technology (website) and the content (treatment) of the telehealth program for engagement. It should be noted that the website usability questionnaire that we used was developed for this study. Our findings were consistent with previous work in this area. Nonetheless, this is a limitation and future work should employ standard evaluations of perceived usefulness and ease of use, such as the System Usability Scale [85].

### Conclusion

Taken together, these data provide support for the potential of telehealth to deliver parent-mediated interventions to parents of children with ASD. Additional research will be important to determine whether the same patterns and predictors of program engagement emerge in larger samples.

## Multimedia Appendix 1

Screenshots of ImPACT Online.

## Abbreviations

ADOS: Autism Diagnostic Observation Schedule
ANOVA: analysis of variance
ASD: Autism Spectrum Disorder
CBT: Cognitive-Behavioral Therapy
CES-D: Center for Epidemiological Studies-Depression Scale
CEWFS: Computer-Email-Web Fluency Scale
DSM-IV-TR: Diagnostic and Statistical Manual – 4th Edition, Text Revision
MANOVA: multivariate analysis of variance
