# Table Correction: Parent Engagement With a Telehealth-Based Parent-Mediated Intervention Program for Children With Autism Spectrum Disorders: Predictors of Program Use and Parent Outcomes

**DOI:** 10.2196/jmir.5246

**Published:** 2015-11-04

**Authors:** Brooke Ingersoll, Natalie Berger

**Affiliations:** ^1^Michigan State UniversityDepartment of PsychologyEast Lansing, MIUnited States

The authors of “Parent Engagement With a Telehealth-Based Parent-Mediated Intervention Program for Children With Autism Spectrum Disorders: Predictors of Program Use and Parent Outcomes” (http://www.jmir.org/2015/10/e227/) have inadvertently reversed the percent of participants completing the program for the self-directed and therapist-assisted group in Table 2 during the final proofreading process. The percent of participants completing the program for the self-directed group should be 69% and the percent of participants completing the program for the therapist-assisted group should be 100%.  Table 2 has now been updated with the correct values in the online version of JMIR, and a corrected version was sent to Pubmed Central. 

