# Correction: Parent Engagement With a Telehealth-Based Parent-Mediated Intervention Program for Children With Autism Spectrum Disorders: Predictors of Program Use and Parent Outcomes

**DOI:** 10.2196/jmir.5329

**Published:** 2015-11-13

**Authors:** Brooke Ingersoll, Natalie Berger

**Affiliations:** ^1^ Department of Psychology Michigan State University East Lansing, MI United States

The authors of “Parent Engagement With a Telehealth-Based Parent-Mediated Intervention Program for Children With Autism Spectrum Disorders: Predictors of Program Use and Parent Outcomes” (http://www.jmir.org/2015/10/e227/) have overlooked errors in participant demographics during the submission and proofreading process. In the Abstract, the sentence “Of the 27 parent participants, the majority were female (26/27, 96%), married (22/27, 81%), with a college degree or higher (18/27, 66%), and less than half were employed outside of the home (10/27, 37%)” should read as follows: “Of the 27 parent participants, the majority were female (26/27, 96%), married (22/27, 81%), with a college degree or higher (*15*/27, *56%*), and less than half were not employed outside of the home (10/27, 37%).” In the Results, the statement “As shown in Table 1, the majority of parent participants were female (26/27, 96%), married (22/27, 81%), with a college degree or higher (18/27, 66%). Less than half of parents were employed outside of the home (10/27, 37%)” should read as follows: “As shown in Table 1, the majority of parent participants were female (26/27, 96%), married (22/27, 81%), with a college degree or higher (*15*/27, *56%*). Less than half of parents were not employed outside of the home (10/27, 37%).” In Table 1, “Employment status (% employed)” should read as follows: “Employment status (% not employed)” and the percent of participants in the study with a college degree should be *56%* instead of 66%. These errors have now been updated with the correct values in the online version of JMIR, and a corrected version was sent to PubMed Central.

